# Intraoperative Localization of Vascular Malformation of Small Bowel by Selective Intra-arterial Dye Injection

**DOI:** 10.4103/1319-3767.70622

**Published:** 2010-10

**Authors:** Priya B. Eshpuniyani, Chetan V. Kantharia, RamKrishna Y. Prabhu, Avinash N. Supe

**Affiliations:** Department of Surgical Gastroenterology, KEM Hospital, Parel, Mumbai, India

**Keywords:** Diffuse lesions, exact localization, methylene blue

## Abstract

Angiomatous malformation is the most common vascular abnormality, accounting for 30-40% cases of obscure GI bleeding from small bowel. Surgical resection is the treatment of choice in severe or recurrent hemorrhage requiring multiple blood transfusions. However, the diffuse nature of the lesions poses a challenge to localize them accurately preoperatively, for exact resection. We present a case in which we have used selective mesenteric angiography with selective cannulation and exact localization of the lesion by injecting dye such as methylene blue, indigo carmine, and fluorescein, to localize the angiomatous malformation before surgical resection and also to determine the exact resection to be done.

Angiomatous malformation is the most common vascular abnormality of the GI tract. Small bowel lesions account for 30-40% cases of obscure GI[1,2] bleeding. These lesions are suspected in patients with a negative upper endoscopy and colonoscopy. In such patients, angiography with selective cannulation with injection of dye is a useful mode of localization of the lesion.[2–5] We report a case of angiomatous malformation in the jejunum in which this method of diagnosis and localization proved to be useful in surgical management.

## CASE REPORT

**Figure 1:**
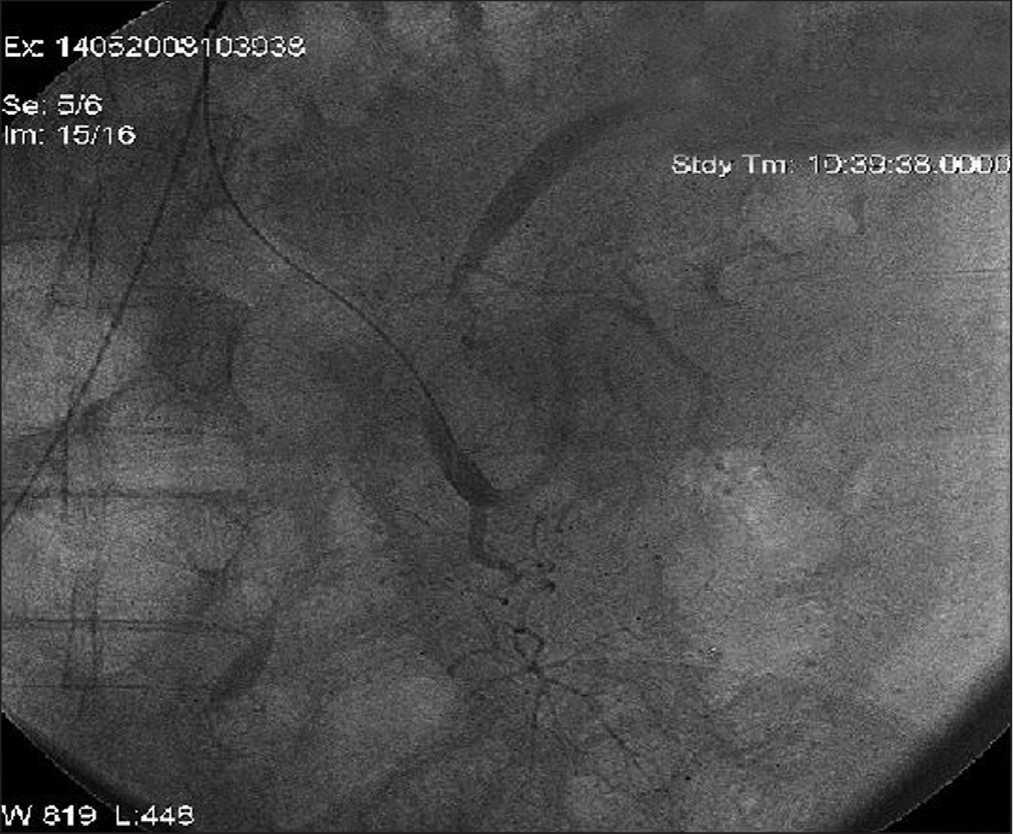
Superior mesenteric angiography with super-selective cannulation into the feeding vessel-showing the diffuse vascular lesion

A sixty year old man presented with malena since 3 months. He had H/O multiple blood transfusions prior to referral. On admission, his vital parameters were stable. Per-abdomen examination, rectal examination, and proctoscopy were normal. His hemoglobin (Hb) was 9 g/dl with rest of the blood investigations being normal. Ultrasonography of the abdomen, upper GI endoscopy, push enteroscopy 1 m from duodeno-jejunal flexure, and colonoscopy were normal. Capsule endoscopy (using M2A capsule) was also normal. Meckel’s scan using 99m-Tc pertechnetate radioisotope and Nuclear Scan using technetium Tc 99m–labeled red blood cells showed abnormal uptake of tracer in the left hypochondrium, extending into the left iliac fossa with the suspicion of active bleeding in the small bowel. The focal nature of the pooling suggested possibility of a vascular anomaly in the proximal jejunum. Triple vessel angiography confirmed angiomatous malformation in the jejunum with the presence of a large jejunal branch arising from the superior mesenteric artery (SMA) [Figure 1] with multiple small micro aneurysms.

**Figure 2:**
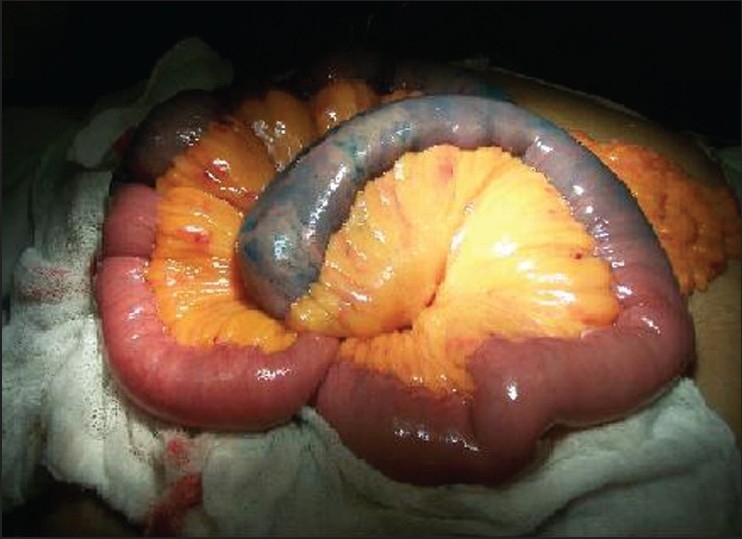
Intraoperative picture showing demarcation of small bowel after injection of methylene blue into the microcathether placed in the feeding vessel

In view of the persistent bleeding, surgery was planned. On the morning of the surgery, triple vessel angiography was done with a 4Fr catheter (Cordis, Florida, USA) placed at origin of SMA and selective canulation of the feeding vessel with a 2.7Fr Prograde microcathether (Teremo, Japan) placed in the feeding vessel. The catheters were secured to the thigh to prevent displacement and the patient was shifted to the operation theatre. On exploration, 1.5 feet of jejunum showed dilated submucosal vessels and increased vascularity. Rest of the bowel appeared normal. Methylene blue was injected into the feeding vessel via the microcatheter following which, more than 1.5 feet was found to be discoloured with a clear demarcation, around 2 cm beyond the abnormal looking bowel on either side [Figure 2]. Resection of the demarcated segment of jejunum was done. The patient did not require any blood transfusion during surgery and postoperatively.

On histopathologic examination, submucosa showed thickened, congested, focally tortuous vascular channels, some being extremely thin walled blood vessels. Findings were consistent with angiomatous malformation.

Post-operative course was uneventful and the patient was asymptomatic at follow up, 4 months postoperatively. His postoperative Hb was 10 mg/dl at last visit.

## DISCUSSION

Angiomatous malformation is the most common vascular abnormality, accounting for 30-40% cases of obscure GI bleeding from small bowel. 15% of lesions are located in jejunum and ileum. Common presentation is malena or hematochezia as bleeding is usually low grade. However, it can be massive in approximately 15% of patients, as seen in our patient.[2]

Surgical resection is the treatment of choice in severe or recurrent hemorrhage requiring multiple blood transfusions.[2] However, exact preoperative localization of the lesion poses a challenge. Various methods such as push enteroscopy, colonoscopy, RBC scan, and selective mesenteric angiography have been used for diagnosis and exact localization of the lesions. Infrequently angiomatous malformations can be detected by visual inspection of the serosal side of the bowel during Laparotomy.[2]

Radionuclide scanning, Tc 99m–labeled red blood cells or ^99m^Tc sulfur colloid, is helpful for detecting and better localization of active bleeding from angiomatous malformation as in our case. Though it detects bleeding with rates as low as 0.1 mL/min, the intermittent nature of bleeding limits the utility of radionuclide studies in angiomatous malformation.[2] Hence, lesions may be missed in patients who are not actively bleeding at the time of presentation, leading to inadequate resection.

Selective mesenteric angiography is a useful diagnostic technique, with its sensitivity ranging from 58 to 86%. The rate of bleeding (0.5 mL/min), technique, and timing in relation to period of bleeding[1,2] are its limiting factors. Thus, there is the possibility of missing out small lesions bleeding slowly or delayed presentation.

In diffuse angiomatous malformation as in our patient, where a single bleeding vessel cannot be identified, routinely used methods such as intraoperative palpation, endoscopy, and visual inspection through multiple enterotomies are of little value, as it does not help to determine the exact extent of the bowel to be resected.

Hence, in such patients, selective mesenteric angiography with selective cannulation and exact localization of the lesion by injecting dye such as methylene blue, indigo carmine, and fluorescein is an excellent modality of investigation and treatment as it assists not only in exact localization of angiomatous malformation before surgical resection,[2–5] but also in determining the extent of resection to be done, by accurate demarcation as in our case. This helps to avoid missing out on lesions which are either diffuse or are not actively bleeding and thus recurrence of the lesion.
